# Dermatophyte Virulence Factors: Identifying and Analyzing Genes That May Contribute to Chronic or Acute Skin Infections

**DOI:** 10.1155/2012/358305

**Published:** 2011-10-04

**Authors:** Rebecca Rashid Achterman, Theodore C. White

**Affiliations:** ^1^Seattle Biomedical Research Institute, 307 Westlake Avenue North, Suite 500, Seattle, WA 98109-5219, USA; ^2^School of Biological Sciences, University of Missouri-Kansas City, 5007 Rockhill Road, BSB 404, Kansas City, MO 64110, USA

## Abstract

Dermatophytes are prevalent causes of cutaneous mycoses and, unlike many other fungal pathogens, are able to cause disease in immunocompetent individuals. They infect keratinized tissue such as skin, hair, and nails, resulting in tinea infections, including ringworm. Little is known about the molecular mechanisms that underlie the ability of these organisms to establish and maintain infection. The recent availability of genome sequence information and improved genetic manipulation have enabled researchers to begin to identify and study the role of virulence factors of dermatophytes. This paper will summarize our current understanding of dermatophyte virulence factors and discuss future directions for identifying and testing virulence factors.

## 1. Introduction

Dermatophytes are the most common cause of fungal infections worldwide, resulting in treatment costs of close to half a billion dollars annually in the USA [1, 2]. The World Health Organization estimates global prevalence of dermatomycoses to be approaching 20% [3]. Despite this, researchers lack a sophisticated understanding of dermatophyte pathogenesis [4]. 

Dermatophytes are the group of filamentous fungi that are the most common cause of cutaneous mycoses. The diseases caused by these organisms are generally named after the part of the body that is infected rather than the infecting organism. For example, tinea pedis refers to athlete's foot and tinea unguium refers to a nail infection. Dermatophyte infections are generally superficial, but immunocompromised patients can experience severe, disseminated disease [5]. Although dermatophyte infections are treatable, there is a high rate of reinfection; it remains to be determined whether this is due to relapse (the fungus not being completely eradicated during treatment) or a new infection [6].

The dermatophytes include three genera of molds in the class Euascomycetes: *Trichophyton*, *Microsporum*, and *Epidermophyton*. Dermatophytes are grouped according to their habitat as being either anthropophilic (human associated), zoophilic (animal associated), or geophilic (soil dwelling). Anthropophilic species are responsible for the majority of human infections; however, species from all three groups of dermatophytes have been associated with clinical disease. Human infections caused by anthropophiles tend to be chronic, with little inflammation, whereas infections caused by geophiles and zoophiles are often associated with acute inflammation and are self-healing [7]. 

The recent sequencing and annotation of several dermatophyte genomes, as well as advances in techniques for genetic manipulation of dermatophytes, provide resources that will aid in elucidating the mechanisms of virulence of these ubiquitous organisms. An understanding of the specific virulence factors that contribute to dermatophyte pathogenesis would aid in the design of effective therapeutics. This paper will summarize the current state of understanding of dermatophyte virulence factors and comment on future directions for their studies.

## 2. Virulence Factor Identification

### 2.1. Virulence Factor Identification Using Bioinformatics Approaches

The sequencing of seven dermatophyte genomes has recently been completed, and the sequences have been made publically available via the Broad Institute database [8]. The Broad Institute sequenced and annotated the genomes of five dermatophyte species. *Trichophyton rubrum* (*Tr*) is anthropophilic and the most common cause of dermatophyte infections in humans worldwide. *Trichophyton tonsurans* (*Tt*) is also anthropophilic and is a major cause of tinea capitis (scalp ringworm). *Tt* was recently found to be present in more than 30% of students in some grade levels at US schools [9]. *Trichophyton equinum* (*Te*) is closely related to *Tt* but is zoophilic and primarily associated with disease in horses. *Microsporum canis* (*Mc*) is also zoophilic and is the most common cause of tinea capitis in Europe [10]. *Microsporum gypseum* (*Mg*) is a geophile that is associated with gardener's ringworm. The strains selected for sequencing are all clinically relevant (associated with human disease) [4] and have been characterized with respect to growth rate, conidiation, and drug susceptibility [11]. Genome sequences of the remaining two species, the phylogenetically related zoophiles *Arthroderma benhamiae *(*Ab*, a teleomorph of *Trichophyton mentagrophytes*) and *Trichophyton verrucosum *(*Tv*), were recently completed by the Hans Knoell Institute (Jena, Germany) and published [12]. These organisms cause a highly inflammatory infection in humans. As expected, comparison of the seven dermatophyte genomes indicates that these species are closely related phylogenetically and each is more closely related to the others than to *Coccidioides immitis* or the dimorphic fungi (unpublished data). This is in agreement with a comparative study of five dermatophyte mitochondrial genomes, which suggested a recent divergence of the dermatophyte clade [13].

All seven genomes were found to encode high numbers of protease-encoding genes compared to related, nondermatophytic fungi ([12] and unpublished data). In particular, dermatophytes appear to have expanded sets of endopeptidases, exopeptidases, and secreted proteases. In contrast, there is little difference in abundance of carbohydrate enzymes of the CAZy family designation [14, 15] between dermatophytes and dimorphic fungi (unpublished data). This highlights the important role of protein degradation in the lifestyle of dermatophytes. 

Secretome analysis of *Ab *during growth on keratin confirmed that proteases made up the largest group of identified secreted proteins [12]. The *Ab *and *Tv* genome sequences also revealed a relatively high number of secondary metabolite gene clusters, and expression of some of these genes were confirmed to be up- or downregulated during keratinocyte infection by *Ab* [12].

As described above, disease caused by human-adapted organisms *Tr* and *Tt* tends to be chronic with low inflammation, whereas zoophiles (*Te*, *Mc*, *Ab*) and geophiles (*Mg*) generally cause an inflammatory infection. The availability of sequence information now allows researchers to use a bioinformatics approach to make predictions about which genes are involved in virulence, as well as differences between species that have adapted to different ecological niches. Preliminary unpublished analysis indicates that among the five genomes sequenced by the Broad Institute, most genes are conserved in all five species, the majority of which are annotated. This is not surprising and confirms the genetic relatedness of the dermatophytes. Of the annotated genes that are unique to a particular species, there does not appear to be any trend. However, there are a number of hypothetical genes unique to each species that potentially play a role in niche adaptation and pathogenicity (unpublished data).

In many pathogenic eukaryotes, including protozoan parasites and fungi, there is a trend for clinical isolates to have cryptic, modified sexual cycles. Although originally thought to be asexual, *Aspergillus fumigatus*, *Candida albicans*, and *Cryptococcus neoformans *are all examples of fungi that cause systemic disease and were shown to have sexual cycles [16, 17]. It is therefore possible that other pathogenic fungi, including anthropophilic dermatophytes, have sexual cycles that have not yet been identified under laboratory conditions. Bioinformatic analysis identified the mating locus in each of the sequenced dermatophyte species, and PCR analysis with additional strains of the species has identified the other mating type, except for *Tr*, where the second mating type has not yet been identified [18]. Recent work has shown normal mating with progeny in the geophilic species *Mg* [19] and the ability of *Tr *to initiate but not complete a mating cycle with a related species of the opposite mating type [20]. Whether *Tr* is able to mate during growth on human skin remains to be determined, and the potential contributions of mating to virulence represent an area of active research. 

Knowledge of the mechanisms of pathogenesis of other fungi also leads to predictions of virulence factors in dermatophytes. For example, the dipeptidyl peptidase DppIV was identified in *Mc* based on sequence similarity [21]. Additionally, dermatophytes have recently been shown to produce melanin or melanin-like compounds, which are predicted to play a role in virulence based on the known role of melanins in other pathogenic fungi [22]. Similarly, *Tr* has been shown to produce xanthomegnin, a toxin previously known to be produced by *Aspergillus*, in culture and during human infection [23]. The complete annotated sequences of dermatophyte genomes will aid in identifying additional putative virulence factors based on sequence similarity, which will then need to be tested experimentally to confirm expression and role during infection.

### 2.2. Virulence Factor Identification Using High-Throughput Screens

The *T. rubrum *Expression Database [24, 25] provided an important starting point for transcriptional analysis by collating expressed sequence tags (ESTs) and transcriptional profiles from microarrays, resulting in the identification of numerous genes involved in growth during a variety of conditions. Complete genome sequencing will enhance our ability to identify open reading frames (ORFs) in future studies. 

Screens have historically been used to identify gene products likely to play a role in virulence. Dermatophytes are known to infect keratinized structures such as skin, hair, and nails, and therefore the ability of dermatophytes to degrade keratin is considered a major virulence attribute. In support of this, a correlation between keratinase activity and pathogenesis has been observed for *Mc* [26] and dermatophytes have been shown to secrete more than 20 proteases *in vitro* when grown in medium containing protein as the sole nitrogen source (reviewed in [27]). As discussed above, genome analysis confirmed expansion of protease genes in the seven dermatophyte genomes ([12] and unpublished data).

Given the importance of keratin to the pathogenic lifestyle of dermatophytes, studies that aimed to identify virulence factors have often examined the response of dermatophytes to growth on keratin. For example, subtractive suppression hybridization (SSH) approaches have been used to compare *Tr* during growth on keratin as compared to glucose [28] or minimal medium [29]. Select genes identified in this manner were confirmed to be upregulated during interaction with keratinocytes [29]. These included a homeobox transcription factor and a zinc-finger protein, which are candidates for acting as transcriptional regulators during infection.

Kaufman et al. found that thioredoxin, cellobiohydrolase, and the protease-encoding gene *Tri m 4* had increased transcription during growth of *T. mentagrophytes *(*Tm*) with keratin as compared to glucose alone [30]. Zaugg et al. constructed a cDNA microarray for *Tr* and examined gene expression during growth on soy and soy + keratin as compared to rich medium (Sabouraud) to find factors induced by one or both proteins [31]. They found that growth in soy or soy + keratin activated a large set of genes encoding secreted endo- and exoproteases, as well as other proteins potentially implicated in protein degradation, some of which appeared to be keratin specific. Interestingly, the authors noted that upregulation of enzymes in the glyoxylate cycle was also observed during growth on soy or soy + keratin, as compared to Sabouraud. The glyoxylate cycle has been implicated in virulence of other microorganisms [32], and its upregulation during dermatophyte growth on keratin was confirmed for *Ab *by Staib et al., who examined gene expression of *Ab *under the same conditions [33]. They found induction of similar sets of orthologous protease-encoding genes as compared to the *Tr* data. 

However, a growing body of evidence suggests that not all keratin-induced proteases play a role during infection. For example, although *Tri m 4 *was induced by the presence of keratin, it was not significantly upregulated when homogenized skin was provided as the sole nutrient source [30]. Furthermore, some of the prominent keratin-induced genes of *Ab* were not found to be strongly upregulated *in vivo *during guinea pig infection and the protease-encoding gene *SUB6* was strongly upregulated *in vivo* but not detectably activated during growth on keratin [33]. Instead, Staib et al. found just one protease-encoding gene, *MCPA*, which was strongly induced during both infection and growth on keratin [33]. Their study identified nonprotease genes, such as those encoding a putative opsin-related protein and enzymes of the glyoxylate cycle, which were upregulated *in vivo*. Likewise, Burmester et al. found that only some of the keratin-induced proteases were strongly expressed during fungus-keratinocyte interaction [12]. Interestingly, they found that secondary metabolites were induced during interaction with keratinocytes. Previous work has shown that antibiotic substances are produced by dermatophytes that may help the fungi compete against bacteria also present on the skin [34, 35]. It is possible that differentially regulated secondary metabolites, perhaps including antibiotics or pigment production, play a role in dermatophyte infection by providing the fungi with an advantage over other microorganisms present on the skin.

Expression of specific secreted subtilisins and metalloproteases was monitored by RT-PCR during *Tr* growth *in vitro* in the presence of keratin, elastin, collagen, or human skin sections [36]. *SUB3*, *SUB4*, and *MEP4* had increased expression under all four conditions (compared to growth in glucose medium). The increased expression of *SUB3* is consistent with *in vivo *findings in *Ab*, although *SUB3* of *Ab *was upregulated only at a low level [12]. Furthermore, *SUB4 *was not found to be upregulated during guinea pig infection by *Ab*.

Together, these results indicate that secreted proteases are not the only virulence factors of dermatophytes and indeed that not all proteases play an overlapping role during infection. Some proteases may be used only during specific stages of infection or might have a more general role in growth that is not specific to virulence. Furthermore, it is possible that each of the dermatophyte species will have a unique program of expression for proteases and other putative virulence factors during infection.

In order to identify additional factors that play a role during infection, investigators have examined the transcriptional response of dermatophytes exposed to environmental factors such as growth on lipids [37, 38], changes in pH [38, 39], and the presence of antifungal drugs [38, 40–45]. They have also assessed transcript abundance during different stages of growth [46–49]. The relationship of these environmental factors to the pathogenesis of dermatophytes is a continuing area of research. 

## 3. Testing the Role of Putative Virulence Factors

### 3.1. Models for Testing Virulence

Although dermatophytes were initially studied in experimental human infections [50], the current most common animal model for studying virulence factors of dermatophytes is the guinea pig [51]. This model has been useful for zoophiles [33, 52–54] but does not provide an accurate infection model for most anthropophilic species [4]. A murine model has been useful for studying the immune response to dermatophytes [55, 56], but again the mice only develop disease in response to infection by zoophiles. An alternative to mammalian models has been to rely on growth of the dermatophyte on keratinized surfaces such as sterilized nail fragments as an indication of pathogenicity [57, 58]. Despite its relative ease, this is a nonquantitative model based solely on the observed (qualitative) ability of the dermatophyte to grow. Its continued use in virulence studies highlights the need for development of a more appropriate model of anthropophilic dermatophyte infection.

*Galleria mellonella* (wax moth) larvae are an established virulence model for several fungal pathogens, including *Candida*, *Cryptococcus*, and the mold *Aspergillus* [59–65]. The *Galleria *model has several advantages as a virulence model, including an immune system with some similarities to the human innate immune system [61, 66]. However, *Galleria* does not appear to be a useful model to study the pathogenic mechanisms of dermatophytes [11].

Recently, researchers have used skin explants as a model for dermatophyte adherence and invasion [67–71]. *Tm* and *Tr* have been tested in this model. Conidial suspensions or pieces of mycelium are applied to a skin explant, and germination and hyphal invasion are monitored microscopically. Dermatophytes can adhere to and invade *ex vivo* skin explants [67–69], and dermatophyte growth is inhibited by antifungal drugs [70]. A reconstructed feline epidermal model [72] and a feline *ex vivo* epidermal model [52] have also been reported for the study of *Mc*, a zoophile whose natural host is cats.

Human epidermal tissues are commercially available, which abrogates the need for researchers to have access to clinical samples and provides a greater degree of standardization between labs that wish to use skin explants as a virulence model. Dermatophyte microconidia are able to germinate and cause damage to these tissues (our own unpublished data). Skin explants therefore represent a possible virulence model to study the initial stages of dermatophyte infection.

### 3.2. Virulence Factor Genes That Have Been Tested

There are a few cases in which a gene hypothesized to play a role in virulence has been deleted or knocked down. Due to the historical difficulties of genetic manipulation of dermatophytes, gene deletions are often not complemented. However, recent genetic advances have provided a foundation for genetic manipulation of dermatophytes [53, 73–77]. Ideally, future studies of dermatophytes should include both a deletion and a complementation of the mutation to definitively prove the role of a gene product in virulence.

A study on the gene encoding malate synthase (AcuE, a key enzyme of the glyoxylate cycle) provided an excellent step towards this goal. *ACUE* was identified as being upregulated during infection of guinea pigs by *Ab* [33]. Grumbt et al. constructed a deletion, Δ*acuE*, and compared its growth to the parental strain on different carbon sources as well as during guinea pig infection [53]. Although they did not see a difference in pathogenicity between the mutant and the wild type, they did see differences in growth when provided with 0.5% olive oil as the sole carbon source, with Δ*acuE* being unable to grow. Complementation with the wild-type allele restored growth. 

*TruMDR2* is a gene identified in *Tr* that is predicted by sequence similarity to encode an ATP-binding cassette (ABC) transporter protein [78]. Deletion of this gene results in increased susceptibility to some antifungal compounds [78]. *TruMDR2 *was found to be upregulated during growth in the presence of keratin as compared to glucose [28, 58], suggesting a role for the transporter protein during infection. To confirm this, the wild-type and deletion mutants were compared for growth on nail fragments. As predicted, the deletion mutant showed a reduced ability to grow [58].

*Tr* also encodes a protein with sequence similarity to *pacC*, a pH-regulated transcription factor in *Aspergillus nidulans* [57]. Disruption of *pacC* results in reduction of keratinolytic activity and a reduction in the ability to grow on nail fragments [57].

As discussed above, *SUB3* has been identified in screens as a putative virulence factor in *Tr* and *Ab. SUB3* expression and activity has also been monitored in *Mc*, where *SUB3* was identified as a 31.5 kDa secreted protein with *in vitro* keratinolytic activity [79]. It was found to be expressed during natural *Mc* infection of cats, although presence of *SUB3* did not correlate to disease state as *SUB3* was found in both symptomatic and asymptomatic infections [79, 80]. Expression of *SUB3* was experimentally reduced using RNA-mediated silencing [21], and the resulting strain was tested in a feline *ex vivo* adherence model [52]. Arthroconidia from the *SUB3* RNA-silenced *M. canis* strain had reduced adherence to feline epidermis compared to the control strain. Although the control-strain arthroconidia did not adhere equally well to epidermis from each of three different cats, for each cat the *SUB3 *RNA-silenced strain had a reduction in adherence that was statistically significant. The strains were also tested for their ability to cause lesions in the guinea pig model, but no difference in virulence was observed [52]. The authors conclude that *SUB3 *is required for adherence to, but not invasion of, the epidermis. It is also possible that the function of other secreted subtilisins masked the loss of *SUB3* or that the guinea pig model does not completely mimic feline infection.

In *Mc*, the *dnr1* gene, which has sequence similarity to nitrogen regulatory genes of other filamentous fungi, is able to complement a loss-of-function nitrogen regulatory mutation (*areA*) in *Aspergillus nidulans*. Disruption of *dnr1 *in *Mc* caused a reduction in the ability of the fungus to grow on medium containing keratin as the sole nitrogen source [81]. A similar result was seen for *Tm* (teleomorph: *A. vanbreuseghemii*) when *tnr1*, a gene with sequence similarity to *areA *and *dnr1*, was disrupted [77]. Neither of these mutants have been studied in a virulence model. Virulence studies of these and other factors identified, for example in screens or through bioinformatics approaches, will be essential to determining the contribution of each factor to disease. 

## 4. The Role of the Immune System

Fungal virulence is the result of interplay between the infecting organism and the host. During dermatophyte infection, cell-mediated immunity is widely considered to be responsible for modulating dermatophyte disease [7, 82–87] and fungal antigens activate T-suppressor and T-helper cells [56]. Differences specific to the host are thought to be important in determining the relative susceptibility of individuals, with factors such as age, gender, and genetics all likely to play a role [85, 87]. These clinical factors will not be reviewed here. 

A recent review of host-dermatophyte interactions is available [83]. We will briefly describe these as they relate to dermatophyte virulence factors. The most numerous cells in the epidermis are keratinocytes, indicating that dermatophytes must primarily interact with these cells. Interestingly, keratinocytes seem to exhibit a differential response following exposure to different dermatophyte species. Tani et al. measured cytokine production by epidermal keratinocytes following coculture with *Tm*, *Tt*, and *Tr* [88]. Of these, *Tm *causes an acute inflammatory response, whereas *Tt *and *Tr* are anthropophiles that cause minimal inflammation. Although all three species induced Interleukin- (IL-) 8 secretion, coculture of keratinocytes with *Tm *resulted in higher levels of IL-8 production than coculture with *Tt* or *Tr*. Additionally, *Tm* but not *Tt* or *Tr* was able to induce secretion of TNF*α.* Similar results were found when cytokine secretion profiles of human keratinocytes were compared during dermatophyte infection with *Ab*, a zoophile and causes a severe inflammatory response, and *Tt* [89]. They found that both species caused an increase in expression of IL-8, which was confirmed by reverse transcriptase-polymerase chain reaction (RT-PCR). However, *Ab* also induced secretion of a broad spectrum of cytokines, whereas *Tt* did not. These studies support the hypothesis that different dermatophyte species induce different immune responses in the host, contributing to the relative severity of the infection. 

Keratinocytes are not the only cells that will interact with the dermatophytes. Phagocytic cells are attracted to the site of infection (reviewed in [87]), and an ability of dermatophytes to survive those interactions would contribute to pathogenesis. Campos et al. determined that *Tr* conidia could germinate in a macrophage, resulting in macrophage death [90]. 

Certainly, it would be advantageous for anthropophiles to downregulate the immune response so as to facilitate chronic infection, and fungal factors that modulate the host immune response represent potential virulence factors. Indeed, mannan extracted from the cell wall of *Tr* inhibited lymphoproliferation of human mononuclear leukocytes *in vitro* [91]. Addition of filtrate solution from *Tm* or *Tr* was shown to induce secretion of IL-1*α*  and basic fibroblast growth factor in keratinocytes [85], with more IL-1*α*  being secreted in cells exposed to the *Tm* filtrate. This suggests that at least some of the putative virulence factors involved in modulating the immune response might be secreted by the fungi. It is likely that cell-associated as well as secreted factors contribute to the dermatophyte's ability to exaggerate or suppress an inflammatory response. 

Temporal expression of proteases has also been postulated to contribute to the relative intensity of the inflammatory response [84]. Recent data comparing secreted proteolytic and lipolytic enzymes in *Tt* and *Te* support this idea [92]. *Tt* and *Te* are adapted to different host species (humans and horses, resp.), and *Tt* causes a mild chronic disease whereas *Te *causes an inflammatory disease in humans. Of the 31 genes studied, each had ≥99.5% sequence identity between the two species; however, transcriptional analysis identified differences in expression during growth on keratin [92]. For example, of the subtilisin-like proteases examined, *Sub6* and *Sub7* had significantly higher expression in *Tt* compared to *Te*, whereas *Sub1 *and *Sub5* had significantly higher expression in *Te* compared to *Tt *[92].

Arthroconidia are produced during some infections and might aid survival in the nail and transmission of the infection to a new host. Arthroconidia can be formed by a majority of *Tr* clinical isolates during growth on nail powder under specific laboratory conditions [93] and have decreased susceptibility to some antifungals compared to microconidia [94]. The precise contribution of arthroconidia to infection and the mechanisms by which arthroconidia interact with host cells remains an area of investigation.

Few studies have examined dermatophyte gene expression in response to human cells [12, 30], and those that have serve to highlight the fact that growth in the presence of keratin does not necessarily reflect conditions during infection. The precise mechanisms by which dermatophyte species interact with host cells at the molecular level remain unknown. There is a clear need to identify the dermatophyte factors involved in pathogenesis, and a logical starting point is to identify dermatophyte genes and proteins that are upregulated during interactions with epithelial cells. To this end, transcriptional and proteomic profiling of dermatophytes during infection of human epidermal tissue, in addition to a bioinformatic approach, may identify additional potential virulence factors. These studies must go further, though. It is imperative that we test the expression and role that these factors play during infection. Only then can we expand our list of true virulence factors of dermatophytes and use the knowledge to inform directions for therapy and preventative measures.
